# Lightweight Long Short-Term Memory Variational Auto-Encoder for Multivariate Time Series Anomaly Detection in Industrial Control Systems

**DOI:** 10.3390/s22082886

**Published:** 2022-04-09

**Authors:** Daniel Fährmann, Naser Damer, Florian Kirchbuchner, Arjan Kuijper

**Affiliations:** 1 Fraunhofer Institute for Computer Graphics Research IGD, 64283 Darmstadt, Germany; naser.damer@igd.fraunhofer.de (N.D.); florian.kirchbuchner@igd.fraunhofer.de (F.K.); arjan.kuijper@igd.fraunhofer.de (A.K.); 2 Department of Computer Science, Technical University of Darmstadt, 64283 Darmstadt, Germany

**Keywords:** anomaly detection, pattern recognition, security

## Abstract

Heterogeneous cyberattacks against industrial control systems (ICSs) have had a strong impact on the physical world in recent decades. Connecting devices to the internet enables new attack surfaces for attackers. The intrusion of ICSs, such as the manipulation of industrial sensory or actuator data, can be the cause for anomalous ICS behaviors. This poses a threat to the infrastructure that is critical for the operation of a modern city. Nowadays, the best techniques for detecting anomalies in ICSs are based on machine learning and, more recently, deep learning. Cybersecurity in ICSs is still an emerging field, and industrial datasets that can be used to develop anomaly detection techniques are rare. In this paper, we propose an unsupervised deep learning methodology for anomaly detection in ICSs, specifically, a lightweight long short-term memory variational auto-encoder (LW-LSTM-VAE) architecture. We successfully demonstrate our solution under two ICS applications, namely, water purification and water distribution plants. Our proposed method proves to be efficient in detecting anomalies in these applications and improves upon reconstruction-based anomaly detection methods presented in previous work. For example, we successfully detected 82.16% of the anomalies in the scenario of the widely used Secure Water Treatment (SWaT) benchmark. The deep learning architecture we propose has the added advantage of being extremely lightweight.

## 1. Introduction

Industrial control systems are used to manage and supervise industrial processes in critical infrastructures. Due to the adoption of internet technologies, ICSs are increasingly being targeted by cyberattacks [1]. This sharp increase in cyberattacks poses a threat to the infrastructures (e.g., electric, water, and natural gas facilities) that are critical for the operation of modern cities. A famous example of a cyberattack targeting an ICS, Stuxnet, is a worm that was discovered in June 2010. Among others, Stuxnet is known to have caused massive damage to Iran’s nuclear program [2].

An ICS is a cyber-physical system (CPS), that is characterized by its high degree of complexity [3]. It typically consists of distributed computing elements, mechanical parts, and electronic parts that communicate via IT network infrastructure, such as the Internet. CPSs are augmenting critical public infrastructure [4] such as transportation, electric power generation, water treatment, and distribution. In the context of Industry 4.0, these systems are increasingly automated, such that they can dynamically adapt to production requirements.

The detection of anomalies in CPSs concerns the identification of unusual system behaviors. The central issue with anomaly detection is to be able to distinguish between normal behaviors and behaviors that are potentially disadvantageous or even dangerous [5]. Although experts can very well define the desired system behavior of a CPS, anomalous events can be anything out of the ordinary and are therefore difficult to recognize. In the industrial domain, connecting devices to the internet is the cause for anomalies such as cyber-attacks or information leaks. Anomalies can also result from malfunctions, operator errors, or software misconfigurations. When an industrial plant is operating efficiently, this may mean that certain safety precautions are taken (i.e., exceptional conditions such as extreme temperatures, pressures, etc., are handled). The analysis of the system behavior and the detection of anomalies in CPSs is quite essential to guarantee the safety and security during systems operation as well as to facilitate CPS maintenance and repair.

The detection of anomalies in CPSs is difficult due to several challenges. When the size of a CPS and its internal dependencies increase, complexity often reaches a point where it is no longer trivial to identify anomalous system behavior. Typically, multimodal monitoring sensors are mixed with actuator states. Water treatment and water distribution applications often involve hundreds of sensors and actuators. For this reason, it is important to develop anomaly detection techniques that can cope with data from various types of sensors and actuators. Another challenge is temporal dependency of sensor and actuator signals. When various plant processes are interdependent, timing plays an important role. This increases the need for solutions that take the temporal nature of sensor signals into account. The use cases we are targeting include intrusion detection, and systems health monitoring, as well as event detection in sensor networks.

**Figure 1:**
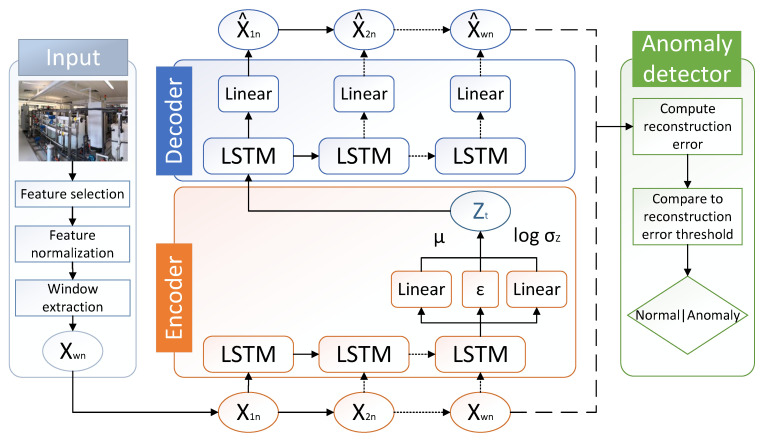
Overview of the methodology proposed in this work. The methodology consits of 3 phases: the input phase (grey), the reconstruction phase (orange, blue), and the anomaly detection phase (green).

We successfully propose the adaption of a variational auto-encoder (VAE) with a long short-term memory (LSTM) [6] network for anomaly detection in CPSs. Frequently, techniques for detecting anomalies directly analyze the raw network communication of the underlying IT infrastructure. In contrast, our proposed method detects anomalies by directly analyzing patterns in sensor measurements and actuator states. Our lightweight long short-term memory variational auto-encoder (LW-LSTM-VAE) architecture is specifically designed to be applied in unsupervised learning scenarios and to cope with various types of sensors and actuators. Figure 1 visualizes the LW-LSTM-VAE architecture proposed in this work.

Our methodology is evaluated against data collected from the SWaT and WADI testbeds, built at the Singapore University of Technology and Design for cybersecurity research [7]. The SWaT testbed is a scaled-down but fully functional water purification plant. Its natural extension, the WADI testbed, is a water distribution system. Our proposed solution proves to be effective in detecting anomalies in these applications. Moreover, it consists of an extremely lightweight architecture with low memory and low training time requirements. Compared to previous works, our proposed method only requires an extremely small amount of model parameters and hidden layers while achieving comparable predictive performance.

An in-depth description of the methodology is presented in Section 3. The experimental setup, including the datasets used for the experiments, data preprocessing, and hyperparameter configurations are described in Section 4. Finally, in Section 5, the results in comparison to previous works are presented, including an analysis that highlights the lightness of our models.

## 2. Related Work

The recognition and prevention of unforeseen events in smart environments has caught the attention of the research community in recent years [8]. Anomaly detection can be understood as a one-class classification problem, where the normal behavior is considered the dominant behavior. Any behavior that is out of the ordinary deviates from the known normal behavior and is therefore considered an anomaly. Deep-learning-based anomaly detection has been proven to perform well, as shown in many surveys [9,10,11,12]. Anomaly detection has also been comprehensively reviewed for intrusion detection in previous literature [13,14,15,16].

Classically, researchers often detected anomalies by directly analyzing data patterns in a reduced space [17] or by decomposing the data into normal, anomaly, and noise subspaces [18]. However, a compressed representation of anomalous data may resemble normal data patterns, such that anomalous data may not be distinguishable from normal data in a reduced space.

Detecting anomalous system behaviors in ICSs is a common problem in smart industrial applications. A variety of different anomaly detection algorithms exist that have been applied in this application domain. The classical anomaly detection algorithms include one-class support vector machines (OC-SVM) [19] and principal component analysis (PCA) [20]. More recently, deep learning algorithms have emerged and have been applied for anomaly detection in ICSs, such as graph neural networks (GNN) [21], Bayesian networks [22], convolutional neural networks (CNN) [20,23,24], generative adverserial networks (GAN) [25,26], auto-encoders (AE) [27] and LSTM [19,28].

In [21], researchers used GNNs to explicitly learn the relationships between sensor and actuator variables in ICSs. Their graph deviation network (GDN) approach learns to detect deviations from normal sensor–actuator relationships. Similarly, a Bayesian network-based anomaly detection strategy was proposed by Lin et al. [22]. Their time automata and Bayesian network (TABOR) approach discovers dependencies between sensors and actuators variables. Deviations from the dependencies are used to recognize abnormal system behaviors. Kravchik et al. [23] proposed the application of one-dimensional convolutional neural networks (1D CNN) for the detection of anomalies. The authors also experimented with an ensemble of CNNs that consider the operational stages of an ICS individually. In [20], Kravchik et al. applied 1D CNN, AE, VAE, and PCA algorithms to the SWaT and WADI benchmark. The authors proposed to perform feature selection based on the Kolmogorov–Smirnov test (K–S test) [29] and considered the time domain as well as the frequency domain of the time-series data.

Reconstruction-based anomaly detection approaches very recently began to be applied in the industrial application domain [24,25,26,27,30]. Reconstruction-based anomaly detection is performed by first transforming the original input data into a low-dimensional representation based on spectral decomposition. The original data instances are then reconstructed from their lower-dimensional representations. After reconstruction, the reconstructed data are compared to the original input data in order to compute a reconstruction error (i.e., the deviation between the original input and the reconstruction of the input). The reconstruction error then directly serves as an anomaly score for the recognition of anomalous data instances [31].

An AE is a representative example of a reconstruction-based approach. It is implemented as a connected network with an encoder and a decoder [32]. For the detection of anomalies in time-series signals (e.g., data recorded by monitoring sensors in industrial facilities), AEs can be applied using a sliding time window [33]. When training an AE with non-anomalous data only, anomalies can be detected based on high reconstruction errors during inference. In [27], Audibert et al. proposed a reconstruction-based anomaly detection approach called USAD, which is based on a combination of two AEs and adversarial training. In [30], Faber et al. proposed a framework to enhance the anomaly detection performance of the USAD model, which was originally proposed by [27]. They also evaluated an auto-encoder-based 1D CNN architecture [34] and an LSTM-VAE architecture [35] on SWaT and WADI. Their framework evolves an ensemble model that is generated based on evolution strategies and involves splitting the sensors and actuators of the facilities into subgroups. In comparison to our proposed LW-LSTM-VAE, the LSTM-VAE architecture originally proposed in [35] requires a significantly higher number of model parameters, while the performance of our models is higher on both benchmarks, as will be presented in Section 5. The method presented in [35] additionally requires to reconstruct the probability distribution of an observation to compute an anomaly score, while our method reconstructs an observation directly. Using the reconstructed probability distribution, their method computes the deviation to the original input distribution to obtain an anomaly score. This is achieved by introducing a progress-based prior of the input data distribution during training, which gradually changes in order to embed the temporal dependency of time-series data into the VAE. Furthermore, in [35], state-based thresholding is introduced, which does have the computational overhead of requiring support vector regression (SVR) to recognize anomalies.

In addition to default AE architectures, VAEs have the potential to abstract over the diverseness of sensor and actuator data by observing statistics of expected distributions [36]. The motivation of using a VAE for anomaly detection is to avoid overfitting by taking into account the statistical distribution of the input and to obtain a more robust latent representation. This is achieved by the means of variational inference. Bayer and Osendorfer [37] used variational inference to estimate the underlying distribution of sequences. Variational inference also opened the way for stochastic recurrent networks, which have been applied to detect robot anomalies [38]. This work proposes a method for anomaly detection in ICSs that is based on a VAE architecture.

For the detection of anomalies in industrial sensor and actuator signals, solutions are needed that take into account the time dependency of the signals. LSTM networks are a suitable choice to capture long- and short-term dependencies of sequential time-series data. There are several advantages of using LSTM networks in comparison to classical approaches (e.g., window approaches or Markov chains). An LSTM-based anomaly detector is expected to have higher representational performance and the memory consumption is expected to be significantly lower when tracking long-term dependencies of sequential time-series data [6]. Additionally, LSTM networks can operate on continuous states. In this work, a VAE architecture is proposed that has been extended using LSTM neural network layers. In the following, previous work that incorporates LSTM networks is presented.

In [28], the authors propose the detection of anomalous events in time series by making use of stacked LSTM neural network architectures. The authors evaluate their method on a subset of the SWaT [7] dataset, but they only considered the first of six operational processes of the SWaT facility. Inoue et al. [19] proposed the application of deep neural networks (DNNs) and OC-SVMs. Their DNN architectures include an LSTM layer to take the time dependence of the sensor values into account. Reconstruction-based methods are presented in [25,26]. Li et al. [26] suggested a GAN-based method for anomaly detection in ICSs. The generator network of their generative adversarial networks-based anomaly detection (GAN-AD) method uses LSTM layers instead of convolutional layers to handle time dependencies. In [25], the same authors propose their multivariate anomaly detection with GAN (MAD-GAN) framework, which is based on GANs and LSTMs for the generator and discriminator. Their MAD-GAN framework outperformed the baseline algorithms the authors report on. However, GANs usually do not perform well when they are applied to small datasets because complex discriminators tend to overfit small datasets. In [24], the authors first extracted features using a stacked denoising auto-encoder (SDA). Features were then predicted by a combination of a 1D-CNN with gated recurrent units (GRU). The statistical deviation between the predicted features and the observed features is used as anomaly score.

Unlike previous works, the methodology proposed in the work, our LW-LSTM-VAE architecture, is a combination of a VAE with an LSTM network, specifically designed for the purpose of anomaly detection in ICSs. Our proposed methodology has the additional advantage of being extremely compact. The LSTM network is used to model the temporal dependencies between sensor and actuator signals. The VAE is used to obtain a more robust model that captures the underlying statistics of the data.

### Dataset Overview

This section provides an overview of related datasets, addressing anomalies in industrial operations. Unlike datasets that are generally considered for the application of anomaly detection algorithms, the availability of datasets that contain sensor and actuator data from ICSs is limited [39]. In the following, a summary of the available industrial datasets that contain sensor and actuator data is provided. The SWaT [7] and WADI [40] datasets that are used for the development and evaluation of our method are described in greater detail. The datasets provided in [41,42] originate from different ICSs. The Power System [41] dataset contains synchrophasor measurements. A synchrophasor is a time-synchronized phasor measurement unit (PMU) that measures the complex amplitude of current and voltage at a given time. The dataset features network traffic logs recorded by the open-source intrusion prevention system (IPS) Snort. Malicious data samples have been generated based on 28 different attack scenarios. The Gas Pipeline [41] dataset contains captured data logs from a gas pipeline supervisory control and data acquisition (SCADA) system.

Remote terminal units (RTU) have been used to monitor the gas pipeline. RTUs are electronic devices that can connect physical devices (e.g., sensors and actuators) to SCADA automation systems. They transfer telemetry data to the systems and/or change the physical state of connected objects based on control messages received from the SCADA system. The dataset has been used to evaluate the ability of learning algorithms to identify data injection attacks. Similarly, the Water Storage Tank [42] dataset focuses on a water storage system.

The Center for Cyber Security Research, iTrust [43], built small-scale but fully functional testbeds that mimic real-world industrial facilities. The testbeds were operated under realistic conditions and datasets have been recorded. The Secure Water Treatment (SWaT) [7], Water Distribution (WADI) [40], and Electric Power and Intelligent Control (EPIC) [44] datasets feature network traffic logs, sensor measurements, and actuator states. The samples contained in the datasets have been annotated to be either normal or anomalous. Anomalous samples have been created by running false injection attacks against the testbeds.

Another dataset originates from the BATtle of the Attack Detection ALgorithms (BATADAL) [45] competition. The competition aims at the proposal of cyberattack detection algorithms for industrial environments. The dataset contains samples recorded in a water distribution network that involves seven storage tanks, eleven pumps, and five valves, controlled by nine programmable logic controllers (PLCs). The network was generated with the epanetCPA toolbox, which allows the injection of cyberattacks and simulates the network’s response to those attacks. The dataset is split into two training sets and a testing set. The training set 1 was generated from a simulation that lasted for one year. It does not contain any attacks; all the data pertain to normal operations. The training set 2 is partially annotated and was recorded over 6 months. It contains several attacks, some of which are approximately annotated. The testing set includes 2089 records with seven attacks. It was recorded over a three-month-long period and was used to compare the performance of the algorithms. From all the discussed datasets, only SWaT and WADI have the desired properties to address the challenges targeted in this work. The SWaT and WADI datasets have a high overall size, high dimensionality, and contain raw data from various types of sensors and actuators, while the fraction of anomalous samples annotated in the datasets is very small.

## 3. Methodology

This section presents the methodology proposed in this work. A visualization of the methodology, including the processing pipeline, is shown in Figure 1. The pipeline of the methodology consists of three main phases: the input phase, the reconstruction phase, and the anomaly detection phase. In the following, we provide an overview of the methodology and describe the particular phases.

In the first phase, the input phase, a dataset is preprocessed. Our methodology involves several preprocessing steps, namely, feature selection, feature normalization, and window extraction.

Feature selection is performed for the individual features of an input dataset since the features do not contribute equally to the anomaly detection task. A feature might be redundant (i.e., a feature separates anomalous from non-anomalous samples less well than another feature) or the distribution of feature values in the training set differs greatly from the distribution of feature values in the testing set. Feature selection is important because it greatly influences the performance of the anomaly detector.

In the feature normalization step, the individual feature values are normalized. Feature normalization has a significant effect on the anomaly detection performance because machine learning algorithms are highly sensitive to varying degrees of feature magnitudes. Section 4.2 describes the feature selection and feature normalization process in further detail.

After feature selection and feature normalization are performed on the input data, windows are extracted from the training and testing sets of the respective datasets. The window extraction step divides time-series data into smaller time-series sub-sequences (i.e., windows) by sliding a fixed size window across the data. A window then consists of multiple samples that are in sequential order over time. Creating windows of samples enables our methodology to cope with the temporal dependency of the sequential time-series data. The window extraction process is further described in Section 3.4. The windows obtained thus serve as input to the reconstruction phase.

In the reconstruction phase, our LW-LSTM-VAE neural network architecture attempts to reconstruct the individual input windows. The intuition behind this is that the algorithm learns to model normality, such that it is able to effectively reconstruct windows that resemble normal data patterns, but it fails at reconstructing windows that resemble anomalous data patterns. After reconstruction, the original input window and the reconstructed window serve as input for the final anomaly detector phase. Further details about the LW-LSTM-VAE architecture are provided in Section 3.3.

In the anomaly detector phase, the deviation between an input window and a reconstructed window is computed. The deviation (i.e., reconstruction error) is used as a measure for normality of a respective window and directly serves as an anomaly score. If the reconstruction error exceeds a defined reconstruction error threshold, a window is classified anomalous, otherwise it is classified normal. Details about the computation of the reconstruction error are provided in Section 3.5. The reconstruction error threshold is described in Section 3.6.

### 3.1. Preliminary: Auto-Encoder

This section describes the foundations of auto-encoders and how sparse auto-encoders (SAE) can be used for reconstruction-based anomaly detection. The anomaly detector presented in this work is based on an AE architecture; therefore, it is important to highlight the fundamental working principles.

An AE performs dimensionality reduction because it reduces the number of input features by combining these features into a reduced number of latent features in the encoding process. An AE assigns each input state to an equivalent point in latent space from where the original input is then derived from its embedded analog in the decoding phase. Formally, a latent space *Z* along with the input data space *X* is defined. An encoder function *E* relates both spaces by representing a mapping from the input space *X* into the latent space *Z* (E(X):X→Z).

A decoder function *D* relates the spaces *Z* and $\hat{X}$ by representing a mapping from the latent space *Z* into the reconstructed input data space $\hat{X}$ ($D\left(Z\right):Z\to \hat{X}$). The decoder function is subject to continuity and therefore returns close data points in the reconstructed input space $\hat{X}$, if the data points are close in the latent space *Z*. The trained decoder *D* reflects the normal data’s distribution. It is possible to find a latent representation $Z_{k}$ for each testing window $X^{test}$ (i.e., anomalous or non-anomalous data) under *D*, where $D(Z_{k})$ denotes the reconstructed testing window. Anomalies are then identified by a deviation from the normal data’s distribution and scored based on the reconstruction error between $X^{test}$ and $D(Z_{k})$.

The intuition behind auto-encoders is that the encoder and a decoder form a bottleneck for the data and are trained in a way that they lose as little information as possible during the encoding/decoding process. With the goal of reducing the reconstruction error in mind, the mean squared error (MSE) loss function is applied during training by gradient descent iterations. The MSE loss function is given by:

(1) $$L_{MSE}=\frac{1}{n}\sum_{i=1}^{n}{\left(X_{i}-\hat{X_{i}}\right)}^{2}.$$

The encoder transformation from *X* to *Z* (E(X):X→Z) and the decoder transformation *Z* to $\hat{X}$ ($D\left(Z\right):Z\to \hat{X}$) are learned in a single process during model training.

### 3.2. Preliminary: Variational Auto-Encoder

This section describes how an AE is extended such that it is capable of capturing the variability of the input data. The latent space of an AE can become irregular due to overfitting, meaning that close data points in the latent space can result in distant data points after applying the decoder function (i.e., render the decoded data meaningless). To address this problem of latent space irregularity, the AE architecture is extended by the means of variational inference. A VAE trains the encoder in such a way that it returns a distribution over the latent space instead of a single data point [46]. To enable the neural architecture to learn a distribution, two dense linear layers are introduced; one layer for the approximation of the mean μ and one layer for the variance σ. The loss function of a VAE is called evidence lower bound (ELBO) and can be derived using the statistical technique of variational inference. The ELBO loss function consists of a reconstruction term and a regularization term. The reconstruction term ensures that the reconstruction error between the input and the output of the network is reduced. It maximizes the probability of obtaining the observed data *x* estimated by the model with parameters θ. The reconstruction term is given by:

(2) $$L_{Reconstruction}=-\log\left(p_{\theta }\left(x\right)\right).$$

The regularization term reduces the distance between the two distributions $q_{\Phi }\left(z|x\right)$ and $p_{\theta }\left(z|x\right)$. The regularization term is given by:

(3) $$L_{Regularization}=D_{KL}\left(q_{\Phi }\left(z|x\right)\|p_{\theta }\left(z|x\right)\right).$$

The complete loss function of our methodology is composed of the reconstruction loss and the regularization loss given by:

(4) $$L=L_{Reconstruction}+L_{Regularization}$$

The optimal parameters of the neural network model are obtained by minimizing the loss function:

(5) $$\theta^{*},\Phi^{*}=\underset{\theta ,\Phi }{arg min}\;L$$

The reparameterization trick is necessary to make the ELBO loss function differentiable [46]. The latent space of the VAE can be considered a set of multivariate Gaussian distributions that is given by:

(6) $$z∼q_{\phi }\left(z|x\right)=N\left(\mu ,\sigma^{2}\right)$$

The equation is modified by the reparameterization trick:

(7) z=μ+σ⊙ε,

where ε∼N(0,I) and ⊙ is the element-wise product. This transformation excludes the stochasticity from the update process. The stochasticity is injected into the latent space through a random vector ε. Using the reparameterization trick, the VAE is trainable [46]. The probabilistic encoder maps a compressed representation of the input into the latent vectors μ and σ.

### 3.3. Our Long Short-Term Memory Variational Auto-Encoder Architecture

This section presents our proposed LW-LSTM-VAE neural network architecture. Our LW-LSTM-VAE is an adaption of a VAE that has been extended using an LSTM network. The LSTM network enables the architecture to cope with the temporal dependency of sensor and actuator signals in sequential time-series data. Our LW-LSTM-VAE neural network architecture attempts to reconstruct the individual windows. The intuition behind this is that the algorithm learns to model normality, such that it can effectively reconstruct windows that resemble normal data patterns, but fails at reconstructing windows that resemble anomalous data patterns. The reconstruction mechanism involves an encoder and a decoder. The encoder performs dimensionality reduction by compressing an input window into a reduced representation in latent space *Z*. The encoder consists of an LSTM layer and two dense layers. The LSTM layer encodes the window, including the temporal dependency between the samples in a window, into a compressed representation. The dense layers map the compressed representation of the input into the latent vectors μ and σ, such that the distribution of the input data is approximated. The ε in the encoder represents the random vector ε for the reparameterization trick that is sampled from a Gaussion normal distribution as shown in Equation (7). The latent representation *Z* is then passed to the decoder to reconstruct the original input window. The decoder consists of an LSTM layer and a dense layer. The LSTM layer in the decoder reconstructs the samples of a window by taking their temporal dependency into account.

If the application is more to classify fixed length sequences, 1D CNNs can usually be trained much faster and perform better. However, in industrial applications, determining an appropriate sequence length is not trivial, as can be seen from the various sequence lengths proposed in previous work. In cases where the data has an unknown length, it is more intuitive to use RNNs than to try to include CNNs. For this reason, we have focused our research on the practical application of LSTMs.

The models we propose require only an extremely small number of model parameters. The main reason for this is that our models make use of LSTM layers in the encoder and decoder; thus, the number of model parameters does not increase with the number of samples in an input window. In the case of fully dense AEs or 1D CNNs, the samples in an input window are usually concatenated; thus, the number of model parameters significantly grows with increasing window sizes. The number of hidden layers used in our models is also significantly smaller compared to the majority of deep-learning methods reported in previous work. In addition, our investigations indicated that a relatively small dimensionality of the latent representation *Z* is sufficient to capture relevant statistics of normal system behavior.

Similar to the default loss function of a VAE, the loss function of our architecture comprises a reconstruction term and a regularization term. The regularization term is given by Equation (3). The reconstruction term we use is the MSE loss function. Minimizing the MSE is equivalent to applying maximum likelihood estimation to our model. The reconstruction term is given by Equation (1). The model parameters are optimized based on the combined loss function given by Equation (4). In the testing phase, the anomalous testing windows are fed into the architecture. The LW-LSTM-VAE architecture then reconstructs the testing windows.

### 3.4. Window Extraction

This section describes how the time-series data are converted. The time series is converted because a suitable data format is required such that it can be further processed by our proposed neural network architecture. Another reason is the temporal dependency between sequential data, which needs to be preserved such that anomalous data patterns can be effectively recognized. The time-series data are converted into individual feature vectors (i.e., windows) using the sliding window method [47]. A window is considered to be a tuple of the form $W_{i}\equiv \langle s_{i},s_{i+1},s_{i+w-1}\rangle$, where $s_{i}$ denotes the *i*-th sample and *w* is the window size. During inference, our LW-LSTM-VAE architecture classifies each window to be either normal or anomalous. The entire time-series dataset consists of *k* samples. By sliding a fixed-size window across the entire time series, k−w+1 windows $W_{1},W_{2},...,W_{k-w+1}$ are extracted. In the training phase, all windows are extracted from the training data. The obtained training windows do not contain any anomalous samples and are passed to the reconstruction mechanism and used for architecture optimization. In the testing phase, all windows from the testing data are extracted including the normal/anomalous label for every sample. Each window is then classified as follows: the entire window is classified as anomalous if the reconstruction error of the window is higher than the reconstruction error threshold; otherwise, the window is labeled normal. The reconstruction error and reconstruction error threshold will be explained in Section 3.5 and Section 3.6, respectively. The normal/anomalous verdict of our anomaly detector is then compared with the ground-truth labels for evaluation. Our anomaly detector labels a window anomalous, regardless of the location of an anomalous sample that occurs in the window. As a consequence, the first occurrence of an anomalous sample can be w−1 samples off. Thus, the temporal resolution of the anomaly detector’s verdicts decreases with increasing window sizes.

### 3.5. Reconstruction Error

This section describes how the deviation (i.e., reconstruction error) between an input window and a reconstructed window is computed. The reconstruction error serves directly as an anomaly score. It measures the degree to which a window resembles normal data patterns. First, the MSE over each feature (i.e., standardized sensor or actuator values) in a window is computed. Second, the mean over the entire window is computed. By computing the MSE over the features of a sample, the individual deviations of each feature are taken into account. This results in an anomaly score for each sample in a window. The degree to which the entire window is anomalous is then measured by taking the mean over the individual reconstruction errors of the samples. The reconstruction error of a window is then given by:

(8) $$Reconstruction\;Error=\frac{1}{W}\sum_{s=1}^{W}\frac{1}{N}\sum_{i=1}^{N}{\left(X_{si}-{\hat{X}}_{si}\right)}^{2},$$

where W denotes the number of samples in a window, N denotes the number of features of a sample, *X* denotes the features of a particular sample, and $\hat{X}$ denotes the reconstructed features. The reconstruction errors of the windows are compared with the reconstruction error threshold to decide whether a processed window is anomalous. The method of setting the reconstruction error threshold is explained in Section 3.6.

### 3.6. Reconstruction Error Threshold

This section describes how the reconstruction error threshold is chosen. The reconstruction error threshold directly serves as a decision function to differentiate between normal and anomalous data.

After model training, the reconstruction errors for the training windows are computed. Only the reconstruction errors obtained from the training windows are used to set the reconstruction error threshold. Since the reconstruction error threshold setting has a large impact on the performance of the anomaly detector, we employ two different ways of setting the reconstruction error threshold: (a) the P-th percentile; (b) the standard deviation from the mean. In the following, both settings are described greater detail.

The P-th percentile (0<P≤100) of a list of N ordered values is the value below which P percent of the observations fall. The P-th percentile is obtained by first calculating the ordinal rank *r* and then taking the value from the ordered list that corresponds to that rank. The ordinal rank r is given by:

(9) $$r=\left\lceil \frac{P}{100}\times N\right\rceil ,$$

where P is the percentile and N is the number of values in the list. Every window extracted from the testing data that results in a higher reconstruction error than the P-th percentile score is considered to be anomalous.

The standard deviation from the mean is computed by taking the mean μ from the training window reconstruction errors and adding the standard deviation σ to the mean. The reconstruction error threshold is then given by:

(10) Threshold=μ+σ.

## 4. Experimental Setup

This section presents the experimental setup. Section 4.1 presents the datasets used for the evaluation of our proposed methodology. The feature selection and feature normalization process is described in Section 4.2. The experimental hyperparameter configurations are described in Section 4.3.

### 4.1. Datasets Utilized in This Work

The SWaT [7] and WADI [40] datasets are the most important for our work. The datasets originate from the SWaT and WADI testbeds, which are small-scale but fully functional testbeds that mimic real-world industrial facilities. The corresponding datasets feature network traffic logs as well as sensor measurements and actuator states. A major reason for our decision to use these datasets for our developments is the application domain. Water treatment and distribution facilities represent critical infrastructure that is important for the operation of a city as well as to maintain the quality of life of its inhabitants. The SWaT and WADI datasets are also very challenging because they have high dimensionality (i.e., a high number of discrete and continuous features), while the fraction of anomalous samples in the testing set is very small. Table 1 lists the properties of the SWaT and WADI datasets. The reported properties include the number of training and testing samples, the dimensionality (i.e., the number of recorded sensors/actuators), and the fraction of anomalous testing samples.

#### 4.1.1. SWaT

The SWaT testbed is a scaled-down but fully operational industrial water purification plant. The SWaT testbed was coordinated with the Public Utility Board of Singapore in 2016. SWaT is capable of producing five gallons of drinking water per minute and it mimics the functionalities of real systems in this field. Table 2 lists the features (i.e., sensors and actuators) of the SWaT dataset. Additionally, the table indicates whether the respective sensor/actuator variables are discrete or continuous. Large-scale urban applications could result from the scientific knowledge gained in the SWaT project [7]. The data collected with the SWaT testbed was accumulated over 11 days. In the last four days of data collection, 36 attack scenarios were executed [48]. The attacks carried out are reflected in the dataset by modified sensor and actuator values. The attacks targeted various attack points, including the physical sensors and actuators, as well as access points to the network communication infrastructure of the CPS (e.g., the attacker sends a malicious command to an actuator). Based on the large number of possible the attack points, 28 attacks focused on a single attack point, while 8 attacks focused on multiple attack points simultaneously. In some cases, the researchers performed the attacks sequentially, and in other cases, they allowed the system to normalize before the next attack was executed. Furthermore, the operational processes of the SWaT testbed are divided into six processes P1–P6. The attacks either targeted a single process or multiple processes of the testbed. For a more detailed description about the attack scenarios, we refer the reader to the original publication [48].

The processes of the SWaT testbed are divided according to their respective operational function. Process P1 manages the supply and storage of the water to be processed. In P2, the water undergoes pretreatment and is tested for quality. P3 consists of an ultrafiltration process in which unwanted substances are filtered out. In P4, the remaining chlorine is removed by dechlorination. In P5, inorganic impurities are reduced by reverse osmosis. Finally, in P6, the purified water is stored for distribution. For our experiments, specifically the neural network parameter optimization of our deep-learning architecture, sensor, and actuator data recorded over seven days of continuous operation is used. Evaluation of our models is performed on the remaining four days of logged data, which include the 36 different attack scenarios. The raw network logs that were provided with the dataset have not been considered.

#### 4.1.2. WADI

The WADI testbed can be considered an extension of the SWaT testbed. Although the WADI testbed is similar to the SWaT testbed, it contains components such as analyzers, booster pumps, and chemical dosing systems [40]. Table 3 list the sensor and actuator features contained in the WADI dataset.

Due to the scale of the numerous pipelines used in real water distribution systems, there can be many possible occasions for anomalies, such as water leakage, congestion, or malicious actuator control. Therefore, scientists built the WADI testbed to be able to simulate attacks and to enable the development of methodologies that can detect these attacks. The training set consists of data that were collected with the WADI testbed for 14 days under normal operating conditions. The testing set was collected during the last 2 days of operation and includes various attacks scenarios. Similarly to the attacks that were carried out on the SWaT testbed, the attacks on the WADI testbed are reflected in the dataset by modified sensor and actuator values and targeted various attack points and operational processes. Additional information about the attacks is provided in the original publication [40].

The WADI testbed is divided into three processes. In P1-W, water delivered from the SWaT testbed is extracted and stored in two tanks. Based on a preset demand pattern that simulates water consumption, P2-W controls the distribution from the storage tanks and six consumer tanks. In P3-W, the water is recycled and returned to P1-W.

### 4.2. Feature Selection and Feature Normalization

This section describes the process of feature selection and data preprocessing for our experiments. Feature selection and data preprocessing is necessary to allow for the removal of unwanted data such that the dataset contains more valuable information after the preprocessing steps. Our proposed reconstruction-based method aims at the reconstruction of the system behavior during normal operation. This is possible if the training data are representative of the testing data. The system’s states and transitions between the states that appear in the training data should also appear in the testing data (i.e., the probability distribution of the training data and the testing data should be similar). Only the samples that were generated by simulating attacks should have a different distribution. For this reason, following the suggestions of [20], feature selection is performed based on the K–S test. The K–S test measures the similarity between the probability distributions of the training and testing data [29]. In [20], Kravchik et al. performed the K–S test between the training and testing set, as well as between the training and validation set, to prove that the validation set serves as a good approximation for the testing set. The authors then removed the features based on the results they obtained on the validation set only because the testing set is supposed to be unknown. Following [20], data from the following sensors has been removed from the respective datasets: AIT201, AIT202, AIT203, P201, AIT401, AIT402, AIT501, AIT502, AIT503, AIT504, FIT503, FIT504, PIT501, PIT502, PIT503 from SWaT; and 1 AIT 001 PV, 1 AIT 003 PV, 1 AIT 004 PV, 1 AIT 005 PV, 2 LT 001 PV, 2 PIT 001 PV, 2A AIT 001 PV, 2A AIT 003 PV, 2A AIT 004 PV, 2B AIT 001 PV, 2B AIT 002 PV, 2B AIT 003 PV, 2B AIT 004 PV, 3 AIT 005 PV from WADI. Additionally, our analysis shows that the variables 2_SV_101_STATUS, 2_SV_201_STATUS, 2_SV_301_STATUS, 2_SV_401_STATUS, 2_SV_501_STATUS, and 2_SV_601_STATUS from the WADI dataset are constant in the training and testing set. Previous work considered these variables for anomaly detection purposes. However, these variables do not contribute to the recognition of the anomalous system states that are annotated in the testing data and, therefore, could be neglected. To demonstrate the generalization capabilities of our proposed method, the constant sensor and actuator variables were also included in our experiments. Furthermore, these variables open up potential attack surfaces and should therefore be taken into account in real applications. According to [48], the system reached stabilization 5–6 h after turning it on. For this reason, the first 21,600 samples from the training data (i.e., normal data) from each dataset were removed, following [25]. Additionally, the features of the training data were normalized by removing the mean and scaling them to unit variance. The features of the testing data were normalized by using the mean and variance of the training data. Following [20], we use 80% of the training data for training and 20% for validation. The testing data (i.e., data annotated with anomaly labels) was not considered during the training phase of our models but for the evaluation of the anomaly detection performance.

### 4.3. Hyperparameter Configuration

This section presents the hyperparameter configurations that were used for the experiments conducted. Table 4 lists the hyperparameter configurations that led to the best-performing instances. In our experiments, we divided the time-series data into smaller time-series sub-sequences by sliding a fixed size window across the data. To investigate the effect on the anomaly detection performance and to find a window size that is sufficient to capture the system dynamics, we experimented with a set of different window sizes, namely *w* = 2, 4, 8, 16, 32. Following [19], our investigation showed that a window size of *w* = 4 samples resulted in the best performing instances.

Additionally, we varied the latent and intermediate layer dimensionality in the range of 16–128. To study the effect of the latent and intermediate layer dimensionality, we provide an ablation experiment including a small version of our architecture, which we refer to as LW-LSTM-VAE-S, and a medium version, which we refer to as LW-LSTM-VAE-M. In the case of the LW-LSTM-VAE-M model, we increased the dimensionality of the latent as well as the intermediate layers and included an additional LSTM layer in the decoder. Table 4 lists the hyperparameters we used for both versions. The particular building blocks of the neural network architectures, the type of the blocks, the output shapes, and the number of required parameters are listed in Table 5 and Table 6 for both architecture versions and datasets, respectively.

The LW-LSTM-VAE neural network architectures were optimized using the Adam optimizer with early stopping enabled. The LSTM layers of the architecture use the rectified linear unit (ReLU) activation function, while the dense output layers use the linear activation function. Experiments were conducted using both the P-th percentile and standard deviation (STD) reconstruction error thresholds introduced in Section 3.6. We set the percentile reconstruction error threshold to the 99th percentile score (denoted by $P_{99\%}$) of the training reconstruction errors.

### 4.4. Algorithm Performance Metrics

This section presents the anomaly detection performance metrics used for the evaluation of the anomaly detection on the SWaT and WADI datasets. The anomaly detection performance is mainly reported using the F1-score performance metric. The SWaT and WADI datasets are highly unbalanced in their class distribution. Thus, the performance metrics reported for our method have to be suitable for unbalanced data. For the evaluations conducted in this work, the anomaly detection performance is reported using the precision (Pre), recall (Rec), and F1-score (F1) metrics that are given by:

(11) $$\begin{array}{c} \mathrm{Precision}=\frac{\mathrm{TP}}{\mathrm{TP}\;+\;\mathrm{FP}}, \end{array}$$

(12) $$\begin{array}{c} \mathrm{Recall}=\frac{\mathrm{TP}}{\mathrm{TP}\;+\;\mathrm{FN}}, \end{array}$$

(13) $$\begin{array}{c} F1=2\cdot \frac{\mathrm{Precision}\cdot \mathrm{Recall}}{\mathrm{Precision}+\mathrm{Recall}}, \end{array}$$

where TP, TN, FP, FN are the numbers of true positives, true negatives, false positives, and false negatives, respectively. Additionally, we report the performance of our models using receiver operating characteristic (ROC) curves, including the area under the curve (AUC) metric.

## 5. Results

This section presents our results on the SWaT and WADI datasets, respectively. To place the achieved results in perspective, we also report all previous works that reported on the used datasets. The results are listed in Table 7. The reported performance metrics include precision (Pre), recall (Rec), and the F1-score (F1).

**Figure 2:**
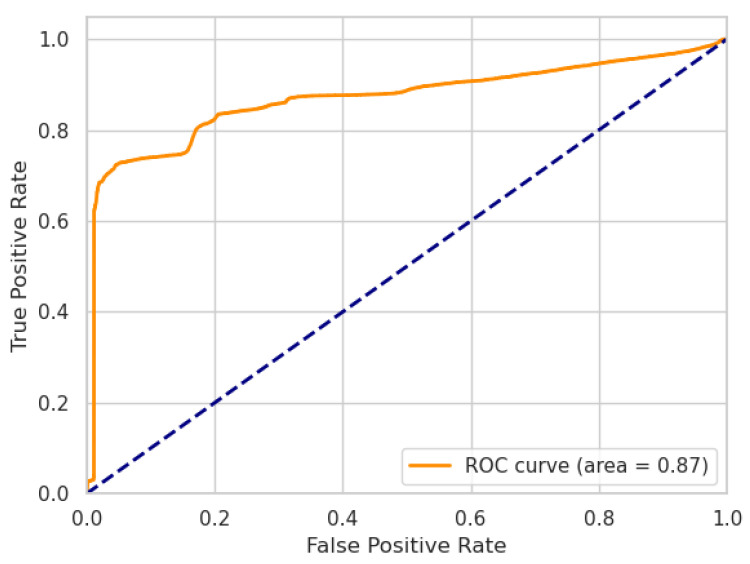
LW-LSTM-VAE-S (SWaT).

**Figure 3:**
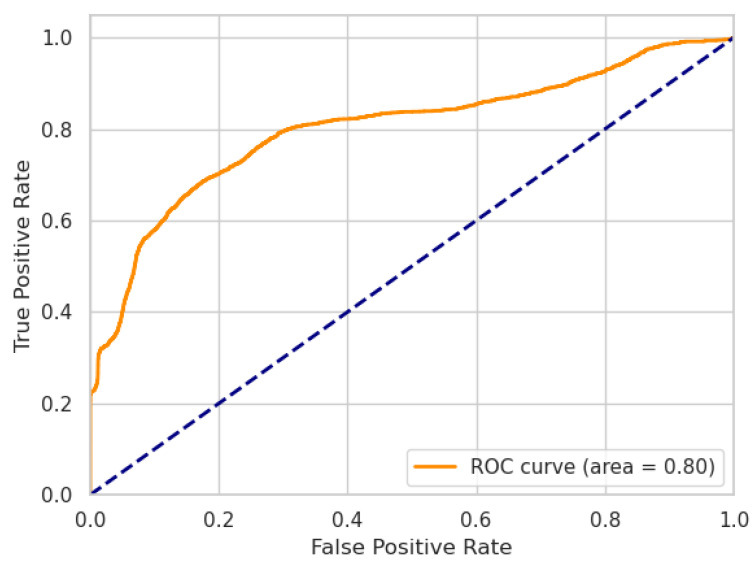
LW-LSTM-VAE-S (WADI).

**Figure 4:**
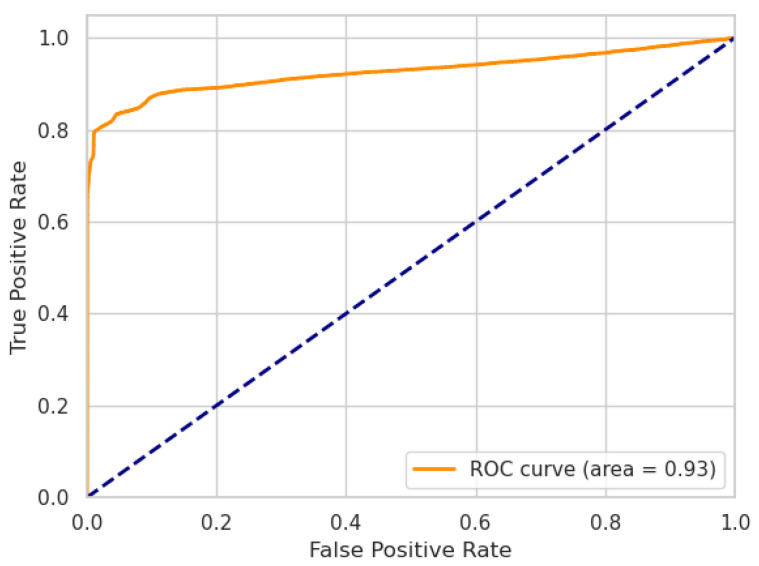
LW-LSTM-VAE-M (SWaT).

**Figure 5:**
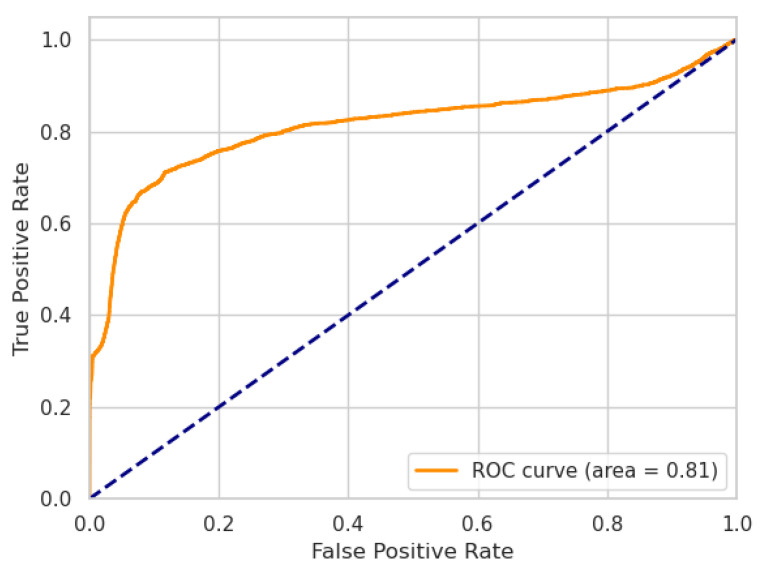
LW-LSTM-VAE-M (WADI).

In the following, we present the results of our ablation experiment. The performances of our LW-LSTM-VAE-S and LW-LSTM-VAE-M models, taking into account the choice of the reconstruction error threshold, are listed in Table 7. In the case that the reconstruction error threshold is determined based on the $P_{99\%}$ method, the LW-LSTM-VAE-S models tend to perform slightly better than the LW-LSTM-VAE-M models. With an F1-score of 78.61%, compared to 77.82%, there is a difference of 0.79 percentage points on SWaT. On WADI, the difference in F1-score is 0.32 percentage points. In the case that the reconstruction error threshold is determined based on the STD method, the LW-LSTM-VAE-M models perform significantly better than the LW-LSTM-VAE-S models. The difference in F1-score is 4.21 and 6.61 percentage points on SWaT and WADI, respectively. Overall, our LW-LSTM-VAE-M models perform better compared to our LW-LSTM-VAE-S models on both datasets. The LW-LSTM-VAE-M models achieve an F1-score of 84.46% on SWaT and 43.72% on WADI. The difference in performance is also indicated by the ROC curves. Figure 2 and Figure 3 show the ROC curves of our LW-LSTM-VAE-S models. The AUC is 0.87 and 0.80 for SWaT and WADI, respectively. In the case of our LW-LSTM-VAE-M models, the AUC is 0.93 and 0.81 for SWaT and WADI, respectively, as shown in Figure 4 and Figure 5.

In the following, we compare the performance of our solution to previous work that reported on the widely used SWat and WADI benchmarks. With 84.46% F1-score on SWaT and 43.72% F1-score on WADI, our LW-LSTM-VAE-M models outperform the majority of the methods reported in previous work.

In [25], Li et al. reported on several classical baseline algorithms, including PCA, K-nearest neighbors (KNN), feature bagging (FB), and AE. In addition to their MAD-GAN framework, the authors also reported on another GAN-based method, namely, efficient GAN (EGAN). Their MAD-GAN framework proved to outperform the reported baseline algorithms by a large margin. The baseline algorithms, namely, PCA, KNN, FB, and AE, also did not perform well on the WADI dataset, which has significantly higher dimensionality compared to the SWaT dataset. In comparison to their MAD-GAN framework, the F1-score of our solution is 7.46 percentage points higher on SWaT and 6.72 percentage points higher on WADI. Our solution outperforms the GAN-AD method presented in [26], in terms of F1-score, by 9.46 percentage points on the SWaT benchmark. The results in [19] indicate that LSTM-based DNNs perform slightly better than OC-SVM models in terms of precision and F1-score. However, the recall and F1-score our LW-LSTM-VAE-M achieves is higher. We outperform their DNN by 4.18 percentage points in terms of F1-score. Additionally, the authors stated that training their DNN architecture with 100 dimensions takes about two weeks on eight NVIDIA Tesla P100s. This indicates a much larger and, thus, less efficient model, in comparison to our LW-LSTM-VAE-M. The training time required by our solution is significantly lower in comparison, as listed in Table 8. The USAD method [27] yields an F1-score of 84.60% on SWaT and 42.96% on WADI. Their method performs similar to our LW-LSTM-VAE-M in terms of F1-score on both datasets. Their method performs 0.14 percentage points better on SWaT. Our method performs 0.76 percentage points better on WADI. With respect to the F1-score, we outperform the methods reported in [30] by 5.46 percentage points on the SWaT benchmark. On the WADI benchmark, only the 1D CNN that the authors optimized using their framework outperforms our solution, by 8.28 percentage points. The model parameter and training time requirements, however, are far above the requirements of our models, as listed in Table 8. Deng et al. [21] applied the DAGMM method and their GDN graph-based method to the SWaT and WADI benchmark. Only on the WADI benchmark does their GDN method outperform our solution, by 13.28 percentage points. On the SWaT benchmark, we achieve better results with respect to the F1-score. The TABOR approach presented in [22] is outperformed by our solution. The method proposed in [24] yields the second-highest F1-score reported in previous work on the SWaT benchmark. With an F1-score of 91.94%, their method outperforms our solution by 7.48 percentage points. However, their method requires computationally expensive preprocessing using an SDA. Kravchik et al. [23] experimented with an ensemble of 1D-CNNs that considers each operational stage of the SWaT testbed individually. The authors report the performance metrics on all data instances and, in addition, on data instances that are annotated as anomalous only. With an F1-score of 92%, their ensemble method ranks among the highest F1-scores on SWaT reported in previous works. However, the F1-score of 92% is reported on anomalous data instances only and does not consider the normal data instances for evaluation. In comparison to their single instance 1D CNN, the F1-score of our solution is 7.86 percentage points higher on all data instances. In [20], the authors applied the learning methods to both the time and frequency domain of the time-series data. They achieved high performance by applying UAEs to the time domain as well as the frequency domain of the time-series data, obtaining F1-scores of 88.50% on SWaT and 75.40% on WADI. However, the authors varied the window sizes and set the reconstruction error thresholds in different ways, depending on the dataset. This is an indicator for overfitting, such that the reported methods might not generalize well on unseen industrial datasets using the same set of hyperparameters. In comparison, our proposed method was evaluated using the same window size and reconstruction error threshold methodologies on both datasets, highlighting its generalization capabilities. Additionally, considering the frequency domain of the time series adds an additional overhead in size and training time requirements to their models.

The discrepancy in the performance of our method compared to previous work is due to several reasons. In [23], the authors used an ensemble of 1D CNNs to consider each operational process of the SWaT testbed individually. The authors stated that a single compact model might not have the representational power to capture the complex operational processes. In [20], the authors included the frequency domain of the time series. Although the addition of the frequency domain adds a significant overhead to their models, the authors justify that particular anomalous data samples can only be recognized in the frequency domain of sensor and actuator signals. In [24], the authors included a more sophisticated, but also a more computationally expensive, means of feature extraction using SDAs. Intuitively, removing noise from sensory signals contributes to better anomaly detection results because physical sensors and actuators have tolerances in measurement accuracy and manufacturing quality. In [21], the authors proposed to explicitly learn the relationships between sensor and actuator variables using GNNs. The explicit graph-based representation of sensor and actuator variables contributed to a more precise depiction of the interrelationships between the individual variables. Deviations in the behavior could then be recognized more precisely, as indicated by the high precision their method achieved.

Overall, the results for the WADI benchmark are worse than for the SWat benchmark, as listed in Table 7. This is because the WADI dataset reflects a more complex and larger system, containing more than twice as many sensor and actuator variables as listed in Table 1. In addition, the proportion of anomalous samples in the testing set is half that of the SWaT benchmark. Furthermore, the testing set of WADI is relatively small compared to the testing set of the SWaT benchmark. Therefore, the small proportion of anomalous samples in the WADI dataset is much more difficult to detect.

Our proposed models do have a significant advantage in terms of memory requirements and training time, because our models only require an extremely small amount of model parameters. Additionally, our method does not involve concatenating the individual features of an input window. In the case of fully dense DNNs or 1D CNNs, the concatenation of the individual features results in significantly larger layer sizes. Instead, our models use LSTM layers that have higher representational power for time-series data. The efficiency of training our LW-LSTM-VAE neural network architecture is shown in Table 8. Unfortunately, the authors of most previous works neither reported the number of model parameters and training efficiency nor provided implementation details sufficient to calculate that precisely. In Table 8, we therefore compare our method to all previous works that specifically reported on the number of parameters the models require. To train the LW-LSTM-VAE models, we used a machine with a Intel Xeon Gold 6130 processor, 256 GB RAM, and an Nvidia GTX 2080Ti-11GB GPU. In the case of the SWaT dataset, training our best-performing LW-LSTM-VAE-M model takes about 38 min for 35 training epochs. In the case of the WADI dataset, training takes about 25 min for 14 training epochs. In [30], Faber et al. report on the number of model parameters and on the training time that the USAD model [27], 1D CNN [34], and the LSTM-VAE originally proposed in [35] require. Regarding the authors, the smallest model they trained on the SWaT dataset, the 1D CNN model, requires 366,476 model parameters compared to the 17,348/90,212 parameters that our LW-LSTM-VAE-S and LW-LSTM-VAE-M models require. Additionally, with a training time of 21/38 minutes on one Nvidia GTX 2080Ti GPU for the respective datasets, our solution only requires a fraction of the training time compared to 16 h for the 1D CNN method on eight Nvidia Tesla V100-SXM2-32GBs. Additionally, we estimated the size of the particular models by using the number of model parameters that the authors report. The number of parameters that our models require is extremely small in comparison.

The detection performance of our proposed method ranks among the best-performing algorithms proposed in previous work. Although not achieving the best recognition performance, we highlight its unique combination of advantages. Based on variational inference, our proposed method learns to capture the variations of sensor and actuator signals. By assuming a simple underlying probabilistic model to describe the data, we enable better latent space organization compared to default AE architectures. We extended the models generalization capabilities and prevented overfitting, such that a more robust anomaly detector is obtained. Additionally, our algorithm is capable of intrusion detection given only past or current data observations. Discrete and continuously valued sensor and actuator signals are treated equally, such that the architecture can easily be adapted to other anomaly detection scenarios. The extremely low memory requirements also point towards future research avenues. Our proposed architecture can potentially be deployed on embedded systems, enabling anomaly detection in other application domains, such as smart buildings or smart homes, where storage requirements and energy consumption play an important role.

## 6. Conclusions

In this work, we propose a reconstruction-based multivariate time-series anomaly detection solution based on our lightweight long short-term memory variational auto-encoder (LW-LSTM-VAE) architecture. Our solution aims at learning the underlying statistics of sensor and actuator data patterns. Our solution is successful in detecting deviations from normal data patterns, such that anomalous system behaviors of CPS are recognized. We considered two real-world datasets for the evaluations conducted. These enable the focus on two applications, anomaly detection in water treatment facilities (e.g., the detection of sensor, actuator fault), and anomaly detection in water distribution facilities (e.g., the detection of intrusions targeting the water supply). Our solution outperforms the majority of methods suggested in previous work in terms of the reported performance metrics. Additionally, our solution is extremely lightweight because the model parameter and training time requirements are extremely low.

## Figures and Tables

**Table 1:** Properties of the SWaT and WADI datasets.

Dataset	Train	Test	Dimensions	Attacks	Anomalies (%)
SWaT [7]	496,800	449,919	51	36	11.98
WADI [40]	1,048,571	172,801	126	16	5.99

**Table 2:** Sensors and actuators of the SWaT testbed.

Type	Device Name	Variable
Flow meter	FIT101, FIT201, FIT301, FIT401, FIT501, FIT502, FIT503, FIT504, FIT601	continuous
Level transmitter	LIT101, LIT301, LIT401	continuous
Analyzer	AIT201, AIT202, AIT203, AIT401, AIT402, AIT501, AIT502, AIT503, AIT504	continuous
Differential pressure transmitter	DPIT301	continuous
Pressure meter	PIT501, PIT502, PIT503	continuous
Motorized valve	MV101, MV201, MV301, MV302, MV303, MV304	discrete
Pump	P101, P102, P201, P202, P203, P204, P205, P206, P301, P302, P401, P402, P403, P404, P501, P502, P601, P602, P603	discrete
Dechlorinator	UV401	discrete

**Table 3:** Sensors, actuators, and controllers of the WADI testbed.

Type	Device Name	Variable
Analyzer indicator transmitter	1_AIT_001_PV, 1_AIT_002_PV, 1_AIT_003_PV, 1_AIT_004_PV, 1_AIT_005_PV, 2A_AIT_001_PV, 2A_AIT_002_PV, 2A_AIT_003_PV, 2A_AIT_004_PV, 2B_AIT_001_PV, 2B_AIT_002_PV, 2B_AIT_003_PV, 2B_AIT_004_PV, 3_AIT_001_PV, 3_AIT_002_PV, 3_AIT_003_PV, 3_AIT_004_PV, 3_AIT_005_PV	continuous
Flow indication transmitter	1_FIT_001_PV, 2_FIT_001_PV, 2_FIT_002_PV, 2_FIT_003_PV, 3_FIT_001_PV	continuous
Level transmitter	1_LT_001_PV, 2_LT_001_PV, 2_LT_002_PV, 3_LT_001_PV	continuous
Pressure meter	2_PIT_001_PV, 2_PIT_002_PV, 2_PIT_003_PV	continuous
Differential pressure transmitter	2_DPIT_001_PV	continuous
Flow totalizer	2_FQ_101_PV, 2_FQ_201_PV, 2_FQ_301_PV, 2_FQ_401_PV, 2_FQ_501_PV, 2_FQ_601_PV	continuous
Modulating valve	2_MCV_101_CO, 2_MCV_201_CO, 2_MCV_301_CO, 2_MCV_401_CO, 2_MCV_501_CO, 2_MCV_601_CO, 2_MCV_007_CO	continuous
Level switch	1_LS_001_AL, 1_LS_002_AL, 2_LS_001_AL, 2_LS_002_AL, 2_LS_101_AH, 2_LS_101_AL, 2_LS_201_AH, 2_LS_201_AL, 2_LS_301_AH, 2_LS_301_AL, 2_LS_401_AH, 2_LS_401_AL, 2_LS_501_AH, 2_LS_501_AL, 2_LS_601_AH, 2_LS_601_AL, 3_LS_001_AL	discrete
Pump	1_P_001_STATUS, 1_P_002_STATUS, 1_P_003_STATUS, 1_P_004_STATUS, 1_P_005_STATUS, 1_P_006_STATUS, 2_P_001_STATUS, 2_P_002_STATUS, 2_P_003_STATUS, 2_P_004_STATUS, 3_P_001_STATUS, 3_P_002_STATUS, 3_P_003_STATUS, 3_P_004_STATUS	discrete
Motorized valve	1_MV_001_STATUS, 1_MV_002_STATUS, 1_MV_003_STATUS, 1_MV_004_STATUS, 2_MV_001_STATUS, 2_MV_002_STATUS, 2_MV_003_STATUS, 2_MV_004_STATUS, 2_MV_005_STATUS, 2_MV_006_STATUS, 2_MV_009_STATUS, 2_MV_101_STATUS, 2_MV_201_STATUS, 2_MV_301_STATUS, 2_MV_401_STATUS, 2_MV_501_STATUS, 2_MV_601_STATUS, 3_MV_001_STATUS, 3_MV_002_STATUS, 3_MV_003_STATUS	discrete
Solenoid valve	2_SV_101_STATUS, 2_SV_201_STATUS, 2_SV_301_STATUS, 2_SV_401_STATUS, 2_SV_501_STATUS, 2_SV_601_STATUS	discrete
Pump speed	2_P_003_SPEED, 2_P_004_SPEED	continuous
Flow indicator controller	2_FIC_101_CO, 2_FIC_101_PV, 2_FIC_101_SP, 2_FIC_201_CO, 2_FIC_201_PV, 2_FIC_201_SP, 2_FIC_301_CO, 2_FIC_301_PV, 2_FIC_301_SP, 2_FIC_401_CO, 2_FIC_401_PV, 2_FIC_401_SP, 2_FIC_501_CO, 2_FIC_501_PV, 2_FIC_501_SP, 2_FIC_601_CO, 2_FIC_601_PV, 2_FIC_601_SP	continuous
Pressure indicator controller	2_PIC_003_CO, 2_PIC_003_PV, 2_PIC_003_SP	continuous

**Table 4:** The hyperparameters we used to obtain the reported results on the respective datasets.

Model	Hyperparameter	SWaT	WADI
LW-LSTM-VAE-S	Window size	4	4
	Window shift (training)	1	1
	Window shift (evaluation)	4	4
	Batch size	128	128
	Learning rate	0.001	0.001
	Intermediate dimension	32	64
	Latent dimension	16	32
	Training epochs	29	11
LW-LSTM-VAE-M	Window size	4	4
	Window shift (training)	1	1
	Window shift (evaluation)	4	4
	Batch size	128	128
	Learning rate	0.001	0.001
	Intermediate dimension	64	128
	Latent dimension	32	64
	Training epochs	35	14

**Table 5:** The neural network architecture of our LW-LSTM-VAE-S models. The particular architecture blocks, the type of the blocks, the output shapes, and the number of required parameters are listed for the respective datasets.

Model	Block (Type)	Input from Block	Output Shape	Parameters
LW-LSTM-VAE-S (SWaT)	1. Input (InputLayer)		(128, 4, 36)	0
	2. Encoder (LSTM)	1	(128, 32)	8832
	3. Encoder μ (Dense)	2	(128, 16)	528
	4. Encoder σ (Dense)	2	(128, 16)	528
	5. Z (Lambda)	3, 4	(128,16)	0
	6. Decoder (Repeat)	5	(128, 4, 16)	0
	7. Decoder (LSTM)	6	(128, 4, 32)	6272
	8. Output (Dense)	7	(128, 4, 36)	1188
	Total number of parameters			17,348
LW-LSTM-VAE-S (WADI)	1. Input (InputLayer)		(128, 4, 112)	0
	2. Encoder (LSTM)	1	(128, 64)	45,312
	3. Encoder μ (Dense)	2	(128, 32)	2080
	4. Encoder σ (Dense)	2	(128, 32)	2080
	5. Z (Lambda)	3, 4	(128, 32)	0
	6. Decoder (Repeat)	5	(128, 4, 32)	0
	7. Decoder (LSTM)	6	(128, 4, 64)	24,832
	8. Output (Dense)	7	(128, 4, 112)	7280
	Total number of parameters			81,584

**Table 6:** The neural network architecture of our LW-LSTM-VAE-M models. The particular architecture blocks, the type of the blocks, the output shapes, and the number of required parameters are listed for the respective datasets.

Model	Block (Type)	Input from Block	Output Shape	Parameters
LW-LSTM-VAE-M (SWaT)	1. Input (InputLayer)		(128, 4, 36)	0
	2. Encoder (LSTM)	1	(128, 64)	25,856
	3. Encoder μ (Dense)	2	(128, 32)	2080
	4. Encoder σ (Dense)	2	(128, 32)	2080
	5. Z (Lambda)	3, 4	(128, 32)	0
	6. Decoder (Repeat)	5	(128, 4, 32)	0
	7. Decoder (LSTM)	6	(128, 4, 64)	24,832
	8. Decoder (LSTM)	7	(128, 4, 64)	33,024
	9. Output (Dense)	8	(128, 4, 36)	2340
	Total number of parameters			90,212
LW-LSTM-VAE-M (WADI)	1. Input (InputLayer)		(128, 4, 112)	0
	2. Encoder (LSTM)	1	(128, 128)	123,392
	3. Encoder μ (Dense)	2	(128, 64)	8256
	4. Encoder σ (Dense)	2	(128, 64)	8256
	5. Z (Lambda)	3, 4	(128, 64)	0
	6. Decoder (Repeat)	5	(128, 4, 64)	0
	7. Decoder (LSTM)	6	(128, 4, 128)	98,816
	8. Decoder (LSTM)	7	(128, 4, 128)	131,584
	9. Output (Dense)	8	(128, 4, 112)	14,448
	Total number of parameters			384,752

**Table 7:** Comparison of our solution to previous work.

Datasets	Methods	Pre	Rec	F1
SWaT	KNN [25]	7.83	7.83	8.00
	FB [25]	10.17	10.17	10.00
	PCA [25]	24.92	21.63	23.00
	EGAN [25]	40.57	67.73	51.00
	AE [25]	72.63	52.63	61.00
	MAD-GAN [25]	98.97	63.74	77.00
	GAN-AD [26]	93.33	63.64	75.00
	OC-SVM [19]	92.50	69.90	79.63
	DNN [19]	98.30	67.85	80.28
	LSTM-VAE [30]	95.69	55.18	72.00
	CNN 1D [30]	95.24	63.73	78.00
	USAD [30]	98.10	66.01	79.00
	DAGMM [21]	27.46	69.52	39.00
	GDN [21]	99.35	68.12	81.00
	TABOR [22]	86.17	78.80	82.32
	USAD [27]	98.70	74.02	84.60
	1D CNN (comb. records) [23]	95.00	65.60	76.60
	1D CNN (ensemble records) [23]	86.70	85.40	86.00
	1D CNN (comb. attacks) [23]	95.00	78.70	86.10
	1D CNN (ensemble attacks) [23]	100.00	85.30	92.00
	PCA (frequency domain) [20]	92.50	72.70	81.50
	VAE [20]	94.00	78.50	85.50
	1D CNN [20]	86.80	85.40	86.10
	UAE [20]	96.50	77.80	86.10
	PCA [20]	92.00	84.10	87.90
	UAE (frequency domain) [20]	91.10	86.00	88.50
	SDA + 1D-CNN + GRU [24]	99.65	83.34	91.94
	LW-LSTM-VAE-S ($P_{99\%}$)	86.84	71.81	78.61
	LW-LSTM-VAE-S (STD)	84.18	76.68	80.25
	LW-LSTM-VAE-M ($P_{99\%}$)	73.91	82.16	77.82
	LW-LSTM-VAE-M (STD)	89.88	79.65	84.46
WADI	KNN [25]	7.76	7.75	8.00
	FB [25]	8.60	8.60	9.00
	PCA [25]	39.53	5.63	10.00
	EGAN [25]	11.33	37.84	17.00
	AE [25]	34.35	34.35	34.00
	MAD-GAN [25]	41.44	33.92	37.00
	USAD [27]	64.51	32.20	42.96
	LSTM-VAE [30]	21.22	29.12	28.00
	USAD [30]	71.24	31.41	43.00
	1D CNN [30]	63.76	43.54	52.00
	DAGMM [21]	54.44	26.99	36.00
	GDN [21]	97.50	40.19	57.00
	PCA [20]	80.70	59.30	68.30
	1D CNN [20]	69.70	73.10	71.40
	VAE [20]	85.30	62.10	71.80
	UAE [20]	91.60	64.00	75.40
	LW-LSTM-VAE-S ($P_{99\%}$)	58.04	31.80	41.09
	LW-LSTM-VAE-S (STD)	41.66	33.45	37.11
	LW-LSTM-VAE-M ($P_{99\%}$)	49.48	34.67	40.77
	LW-LSTM-VAE-M (STD)	72.71	31.25	43.72

**Table 8:** The training efficiency of the lightweight neural network architectures presented in this work. Unfortunately, only [30] reported the specific model parameters for direct comparison with previous works. * The model size is computed based on the Float32 data type. ** The memory consumption refers to a batch size of 128 that we used for training our models.

Model	Parameters	* Model Size	** Training Memory Consumption	Training Time
LSTM-VAE (SWaT) [30]	2,378,496	∼ 9291 KiB	-	24 h
USAD (SWaT) [30]	3,937,360	∼ 15,380 KiB	-	32 h
1D CNN (SWaT) [30]	366,476	∼ 1431 KiB	-	16 h
LW-LSTM-VAE-S (SWaT)	17,348	67.77 KiB	347.77 KiB	21 min
LW-LSTM-VAE-S (WADI)	81,584	318.69 KiB	1038.69 KiB	13 min
LW-LSTM-VAE-M (SWaT)	90,212	352.39 KiB	52,712.39 KiB	38 min
LW-LSTM-VAE-M (WADI)	384,752	1502.94 KiB	116,542.94 KiB	25 min

## Data Availability

Publicly available datasets were analyzed in this study. These data can be found here: https://itrust.sutd.edu.sg/itrust-labs_datasets (accessed on 6 October 2020).
